# CXCR7 Is Highly Expressed in Acute Lymphoblastic Leukemia and Potentiates CXCR4 Response to CXCL12

**DOI:** 10.1371/journal.pone.0085926

**Published:** 2014-01-31

**Authors:** Rita de Cássia Carvalho Melo, Ana Leda Longhini, Carolina Louzão Bigarella, Mariana Ozello Baratti, Fabiola Traina, Patrícia Favaro, Paula de Melo Campos, Sara Teresinha Olalla Saad

**Affiliations:** 1 Centro de Hematologia e Hemoterapia, Universidade de Campinas, Campinas, São Paulo, Brasil; 2 Departamento de Ciências Biológicas, Universidade Federal de São Paulo, Diadema, São Paulo, Brasil; University of Florida, United States of America

## Abstract

Recently, a novel CXCL12-binding receptor, has been identified. This CXCL12-binding receptor commonly known as CXCR7 (CXC chemokine receptor 7), has lately, based on a novel nomenclature, has received the name ACKR3 (atypical chemokine receptor 3). In this study, we aimed to investigate the expression of CXCR7 in leukemic cells, as well as its participation in CXCL12 response. Interesting, we clearly demonstrated that CXCR7 is highly expressed in acute lymphoid leukemic cells compared with myeloid or normal hematopoietic cells and that CXCR7 contributed to T-acute lymphoid leukemic cell migration induced by CXCL12. Moreover, we showed that the cellular location of CXCR7 varied among T-lymphoid cells and this finding may be related to their migration capacity. Finally, we hypothesized that CXCR7 potentiates CXCR4 response and may contribute to the maintenance of leukemia by initiating cell recruitment to bone marrow niches that were once occupied by normal hematopoietic stem cells.

## Introduction

Chemokine receptors belong to the superfamily of heptahelical G protein-coupled receptors (GPCRs) and are involved in a vast array of physiological events [1]–[3]. Among 18 known chemokine receptors, lies CXCR4 whose cognate ligand is CXCL12. CXCL12 is well known to represent the major chemokine for initiating stem cell migration [4], [5]. The majority of cytokines that mediate stem cell migration do so via modulation of either CXCL12 or CXCR4 [6]. Thus, the CXCL12/CXCR4 axis has been identified as the central axis for stem cell mobilization from the bone marrow and for homing to ischemic tissues [5]–[16]. To date, most studies addressing the involvement of chemokines and their receptors in leukemic cell tropism have concentrated on the interaction of CXCL12 and its receptor CXCR4. Given that bone marrow (BM) stromal cells are major producers of CXCL12 [17], [18] and that CXCR4 expression is thought to be higher in BM-residing blasts than in circulating blasts, CXCL12/CXCR4 interactions are likely to facilitate the retention of blasts in the BM [18], [19]. Recently, another CXCL12-binding receptor has been identified. This receptor is more commonly known as CXCR7 but lately, based on a novel nomenclature, has received the name ACKR3 [3], [4], [14], [15], [20]–[23]. It has high affinity to CXCL12 and CXCL11, however, unlike chemokine receptors (GPCRs), CXCR7 is an atypical chemokine receptor and is not G_i_-protein-coupled and does not affect Ca^+2^ mobilization [3], [4], [15], [23]–[25] due to modifications in the Asp-Arg-Tyr-Leu-Ala/Ile-Val (DRYLA/IV) motif [26], [27], [28], but may act as a β-arrestin-biased receptor [23], [29], [30] and/or as a chemokine scavenging receptor for CXCL12 and CXCL11 [16], [29], [31]. In human tissues, CXCR7 expression has been described in active tumor-associated endothelial cells (ECs) and in many types of tumors, and has been shown to be essential for the survival and growth of tumor cells [3], [11], [15], [20], [23], [32], [33]. Growing evidence indicates a role for CXCR7 in cancer cell proliferation and migration, however little is known as to the contribution of this binding receptor to CXCL12– mediated effects [14], [22], [34], [35]–[37]. It is widely accepted that all CXCR7-dependent signaling may depend on different cellular contexts and types. Direct signaling and/or chemokine responses of CXCL12 and CXCL11 through CXCR7 have been shown to be β-arrestin protein coupled and to activate kinase phosphorylation, leading to increased motility and chemotaxis [23], [26], [38].

The relative expression levels of CXCR4 and CXCR7 could be critical in determining cell response to CXCL12 [14]. Heterodimerization between CXCR4 and CXCR7 has been postulated to be a mechanism for modulating CXCR4 function [14], [25], [30], [35], [39]. Furthermore, co-expression of CXCR7 with CXCR4 resulted in the modulation of CXCR4-mediated Gi activation and signaling. In addition, Décaillot et al. demonstrated that the CXCR4-CXCR7 complex constitutively recruits β-arrestin leading to increased cell migration of CXCR4-expressing breast cancer cells [3]. Given that CXCL12/CXCR4 signaling is deregulated in patients with myelodysplastic syndromes (MDS) and leukemias [26] and the recent discovery of CXCR7 as an additional receptor for CXCL12, the aim of the present work was to investigate CXCR7 expression and function in MDS and leukemias, and to elucidate whether CXCR7 affects CXCR4 response to CXCL12 in these malignances.

## Materials and Methods

### Bone Marrow and Peripheral Blood Cells

Bone marrow (BM) samples, collected from 12 healthy donors, 39 MDS, 23 Acute Myeloid Leukemia (AML) and 11 from Acute Lymphoblastic Leukemia (ALL) patients, classified based on the World Health Organization (WHO) system (range 20–85 years, median age 62.5 years), were analyzed. All patients that attended the clinic between 2006 and 2011, with a confirmed diagnosis of MDS, AML or ALL and who where untreated at the time of the study were included. Peripheral blood samples were collected from 4 healthy donors. All healthy controls and patients provided informed written consent and the study was approved by the ethics committee of the University of Campinas. Patients’ characteristics are described in Table 1.

**Table 1:** Clinical characteristics of patients.

Characteristics	Value
**Age ** ***y*** **, median (range)**	62.5 (20–85)
**Sex, ** ***n***	
**Male/Female**	37/36
WHO classification, *n*	
RCDU	0
RCMD	21
RARS	2
RAEB1	8
RAEB2	8
AML	23
T-ALL	7
B-ALL	4

RCDU indicates refractory cytopenia with unilineage dysplasia; RCMD, refractory cytopenia with multilineage dysplasia; RARS, refractory anemia with ring sideroblasts, RAEB1/2, refractory anemia with excess blasts 1/2.

### Cell Lines and Antibodies

The human cell lines used in this study were U937, P39, K562, KG -1, Daudi, Raji, MOLT4 and Jurkat. All cell lines were obtained from ATCC, Philadelphia, PA. The cells were cultured in RPMI 1640 (Gibco–Invitrogen), supplemented with 100 µg/mL streptomycin, 63 µg/mL penicillin and 1 mL fungizone (Gibco-Invitrogen) in the presence of 10% heat inactivated fetal bovine serum (FBS; Vitrocell Embriolife) in a humidified atmosphere at 37°C in 5% CO2 and used between 5 and 15 passages.

The mAb anti-CXCR7 (ab72100) was from Abcam apl (Cambridge, MA, USA), mAbs anti-Op18 (sc-55531) and anti-E-cadherin (sc-8426) and the polyclonal antibodies anti-Fusin (sc-6190), anti-β-actin (sc-1616) and anti-GAPDH (sc-32233) were from Santa Cruz Biotechnology (Santa Cruz, CA, USA). The anti-rabbit-HRP and anti-goat-HRP secondary antibodies were from KPL (Kierkegaard & Perry Laboratories, Inc; Gaithersburg, MD, USA) and Alexafluor® 488-conjugated anti-rabbit, Alexafluor® 633-conjugated anti-goat and Alexafluor® 555-conjugated anti-mouse secondary antibodies were from Molecular Probes® (Leiden, The Netherlands). (PE) anti-human/mouse CXCR7 clone 8F11-M16 and (PE) mouse IgG2b, κ isotype ctrl were acquired from Biolegend (San Diego, CA, USA).

### Flow Cytometry

Expression of CXCR7 in U937, P39, K562, KG -1, Daudi, Raji, MOLT4 and Jurkat cells was evaluated by FACS analysis. Briefly, 1×10^6^ cells were collected, washed with phosphate-buffered saline (PBS), incubated with 10 µg/mL anti-CXCR7 for 20 min, at room temperature, in the dark, and then resuspended in 200 µL of 1% paraformaldehyde. For intracellular staining, the cells were fixed with 100 µL of 4% paraformaldehyde (10 min, room temperature), permeabilized with 100 µL of permeabilizing solution containing 0.2% BSA, 0.1% azide, 0.5% saponin dissolved in PBS and then labeled and resuspended as described above. Fluorescence cell analysis was performed with a FACSCalibur (Becton–Dickinson, CA, USA).

**Figure 1:**
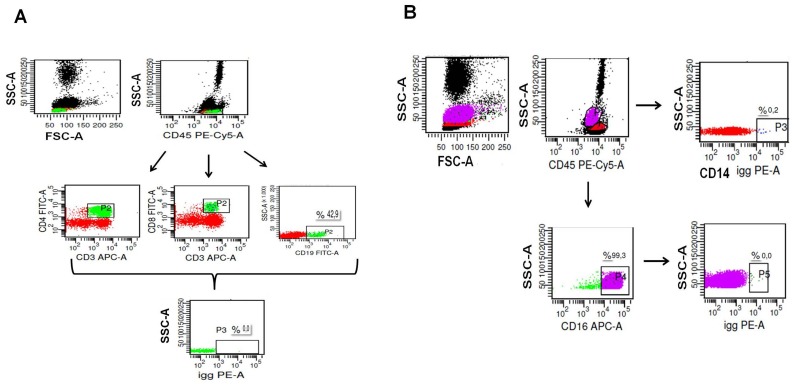
Schematic model of flow cytometric analysis. A) An FSC/SSC gate and anti-CD45^+^/SSC was created around the viable lymphocyte population for further analysis of CD3^+^CD4^+^, CD3^+^ CD8^+^ subsets and CD19^+^ cells. B) An FSC/SSC and anti-CD45^+^/SSC gates were created around the viable granulocyte population for further analysis of CD14^+^ cells and CD16^+^ cells.

To evaluate the expression of CXCR7 in definitive (adult) human leukocyte subsets, peripheral blood was collected, erythrocytes were lysed and the remaining cells were stained with the conjugated mAb (Pe-Cy5) anti-CD45, (FITC) anti-CD14, (APC) anti-CD16, (APC) anti-CD3, (FITC) anti-CD4, (FITC) anti-CD8 and (FITC) anti-CD19. An FSC/SSC gate was created around the viable lymphocyte population for further analysis of CD19^+^ cells, CD3^+^CD4^+^ and CD3^+^ CD8^+^ subsets (Figure 1A). FSC/SSC and anti-CD45^+^/SSC gates were created around the viable granulocyte population for further analyses of CD14^+^ and CD16^+^ cells, as designed in Figure 1B. Data acquisition was performed using a FACScalibur Flow Cytometer (Becton Dickinson, Franklin Lakes, Nj) and analyses were carried out using CellQuest and BD FACSDiva software (Becton Dickinson, Franklin Lakes, Nj).

### Real-time RT-PCR Analysis

Total BM cells of individuals (healthy donors, MDS, AML and ALL patients) and U937, P39, K562, KG -1, Daudi, Raji, MOLT4 and Jurkat cells were submitted to RNA extraction using Trizol®, following the manufacturer’s instructions (Invitrogen, Carlsbad, CA, USA). The reverse transcription reaction was performed using ReverdAid™ First Strand cDNA Synthesis Kit, according to the manufacturer’s instructions (MBI Fermentas, St. Leon – Rot, Germany). Expression of CXCR7 mRNA was detected by Maxima Sybr Green qPCR Master Mix, following the manufacturer’s instructions (MBI Fermentas, St. Leon – Rot, Germany) in the ABI 7500 Sequence Detection System (PE – Applied System) using specific primers: forward 5′-GGC TAT GAC ACG CAC TGC TA-3′, reverse 5′-CTC ATG CAC GTG AGG AAG AA-3′. HPRT and GAPDH were used as endogenous controls and the primers were respectively: forward 5′-GAA CGT CTT GCT CGA GAT GTG A-3′, reverse 5′-TCC AGC AGG TCA GCA AAG AAT-3′ and forward 5′-GCA CCG TCA AGG CTG AGA AC-3′, reverse 5′-CCA CTT GAT TTT GGA GGG ATC T-3′. Three replicates were run on the same plate for each sample and a negative “No Template Control” was used for each primer pair. Relative levels of gene expression were quantified using the equation, 2^−ΔΔCT^ [40].

### CXCR7 Silencing in MOLT4 and Jurkat Cell Lines

MOLT4 and Jurkat cells were transduced with lentivirus-mediated shRNA cop *GFP* control (sc-108084) or lentivirus-mediated shRNA targeting *CXCR7* (sc- 94573-V) from Santa Cruz Biotechnology (Santa Cruz, CA, USA) and are herein named shControl and shCXCR7 cells, respectively. Briefly, 2×10^5^ MOLT4 and Jurkat cells were transduced with lentiviral particles by spinoculation and were selected using 0.3 µg/mL and 0.75 µg/mL of puromycin, respectively, for 2 weeks. The efficiency of CXCR7 silencing was verified by western blot using a specific antibody for CXCR7.

### *In vitro* Treatment of Cell Lines with Antagonist of Receptor CXCR4 (AMD3100)

MOLT4 (shControl and shCXCR7) and Jurkat cells (shControl and shCXCR7) were incubated with 1.25 µg/mL AMD3100 (Sigma-Aldrich®, St. Louis, MO) for 1 hour and then submitted to transwell migration assay.

### Western Blot

Equal amounts of protein of total extracts obtained from cell lines were submitted to SDS – PAGE and Western Blot analysis with specific antibodies and ECL™ Western Blot Analysis System (Amersham Pharmacia Biotech, UK Ltd., Buckinghamshire, England). Quantitative analyses of optical intensity protein bands were determined with Un-Scan-it gel – Version 6.1.

### Confocal Florescence Microscopy

Confocal imaging was carried out using primary antibodies against CXCR7, CXCR4, E-cadherin, Op18 and Alexafluor 488-conjugated anti-rabbit, Alexafluor 633-conjugated anti-goat and Alexafluor 555-conjugated anti-mouse secondary antibodies. MOLT4 and Jurkat cells were immobilized on cover slips previously treated with poly-L-lysine (1 mg/mL), fixed with 4% paraformaldehyde-PBS for 15 min and permeabilized in PBS-0.5% Triton-X-100 for 10 min. The cells were blocked with 3% skimmed milk-PBS and then incubated with the indicated primary (overnight, 4°C) and secondary (2 h, room temperature) antibodies. Slides were mounted using the ProLong Gold antifade reagent with DAPI (Molecular Probes®, Leiden, The Netherlands) and examined in the National Institute of Science and Technology on Photonics Applied to Cell Biology (INFABIC) at the University of Campinas, using a Zeiss LSM 780-NLO confocal on an Axio Observer Z.1 microscope (Carl Zeiss AG, Germany). Images were collected using 1024×1024 image format and 63×optical zoom. In the absence of primary antibodies, staining of secondary antibodies (negative controls) failed to produce any significant staining.

### Transwell Migration Assay

MOLT4 (shControl and shCXCR7) and Jurkat cells (shControl and shCXCR7) treated or not with AMD3100 (1.25 µg/mL) were submitted to migration assay performed as previously described [41]. Briefly, polycarbonate membranes were incubated with 1 mg/mL of poly-L-lysine in dd-water for 1 h at 37°C and then washed twice with water. The cells were washed twice with RPMI containing 0.1% BSA, then seeded at a density of 5×10^5^ cells into the upper chambers of Transwell inserts (5 µM pore size, Costar Transwell; Corning Costar Corning, NY, USA) and allowed to migrate for 4 h. Medium with 0.1% BSA and medium or 0.1% BSA containing CXCL12 (200 ng/mL) in the lower compartment of the transwells were used as negative control and chemoattractant, respectively, as previously described [42]. The number of migrated cells was counted and was expressed as a percentage of the input, i.e., the number of cells applied directly to the lower compartment in parallel wells. The migration of cells was normalized to 100% +/− sd of triplicates as previously described in our laboratory [43].

### Metylthiazoletetrazolium (MTT) Assay

Cell proliferation was measured by MTT assay. MOLT4 (shControl and shCXCR7) and Jurkat cells (shControl and shCXCR7) were serum-starved in 0.5% FBS for 12 hours. A total of 5×10^4^ cells per well were then plated in a 96-well plate in RPMI 10% FBS. In brief, 10 µL of a 5 mg/mL solution of MTT were added to the wells and incubated at 37°C for 4 hours. The reaction was stopped by using 100 µL of 0.1 N HCl in anhydrous isopropanol. Cell growth was evaluated by measuring the absorbance at 570 nm, using an automated plate reader. All conditions were tested in six replicates.

### UV-induced Apoptosis Assay

A total of 5×10^5^ cells of MOLT4 (shControl and shCXCR7) and Jurkat (shControl and shCXCR7) were seeded on 6-well plates and were exposed to a dose of 10 J/m^2^ for different periods of time (0, 3 and 6 hours). Cells were then washed twice with ice cold PBS and resuspended in binding buffer containing 1 µg/mL PI and 1 µg/mL FITC labeled annexin-V. All specimens were analyzed on a FACSCalibur after incubation for 15 minutes at room temperature in a light-protected area. Ten thousand events were acquired for each sample.

### Statistical Analysis

The comparison of the relative expression of CXCR7 among different patient groups, among peripheral blood leukocyte subsets and the results of migration assays were analyzed using *Mann-Whitney* test. Two-tailed *Spearman*’*s* correlation coefficient was also used. These tests are available in GraphPad Prism version 5.0. P value <0.05 was considered statistically significant.

## Results

### CXCR7 mRNA has a Higher Expression in BM Samples of ALL Patients

**Figure 2:**
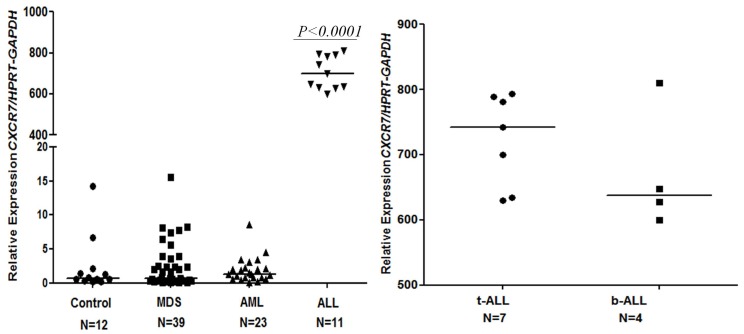
CXCR7 has higher expressed in ALL. Quantitative expression of *CXCR7* mRNA in patient cells relative to healthy donor cells and among different patient groups. Real time RT-PCR was performed on cDNA from the samples of patients with hematopoietic malignancies or from bone marrow samples from healthy donors. Each dot indicates the relative CXCR7 expression for each patient. Horizontal lines represent medians. mRNA expression levels of *CXCR7* were normalized by *HPRT* and *GAPDH* endogenous control. A) *CXCR7* mRNA was highly upregulated in BM samples from ALL patients compared to normal hematopoietic cells samples (*P<0.0001*) and to MDS and AML patients samples (*P<0.0001*). There was no significant difference in *CXCR7* expression among patients with MDS, AML, and normal hematopoietic cells. B) Among ALL-diagnosed patients, *CXCR7* expression was more pronounced in the T-ALL subtype; *Mann-Whitney* test.

**Figure 3:**
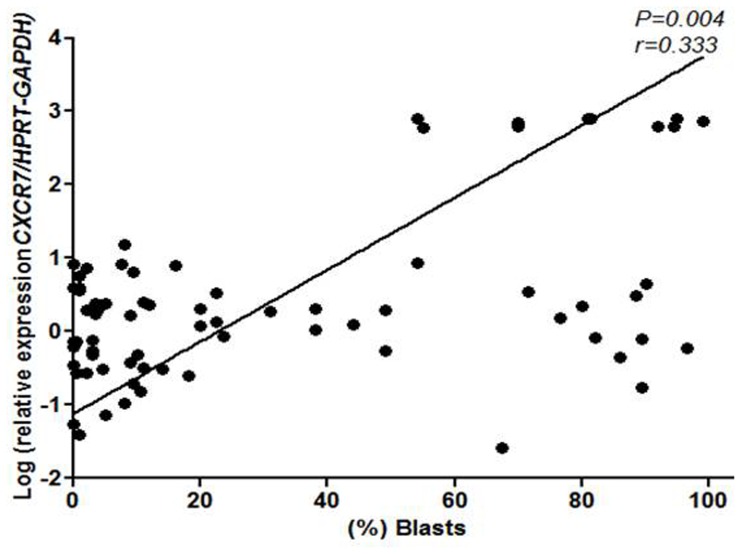
CXCR7 positively correlates with the percentage of blasts in the bone marrow. Correlation of log-transformed relative expression of *CXCR7/HPRT-GAPDH* and the percentage of blasts in the bone marrow of MDS, AML and ALL patients showed *CXCR7* expression levels to be positively correlated with bone marrow blast counts (*P = 0.004*). Two-tailed *Spearman*’*s* correlation. The number of individuals is shown in the figure.

We investigated the *CXCR7* gene expression profile on a sample of patients with hematopoietic malignancies in comparison to bone marrow samples from healthy donors. Interestingly, *CXCR7* mRNA was highly upregulated in BM samples from ALL patients compared to normal hematopoietic cell samples (0.68 [0.17 to 14.1] versus (vs.) 700.2 [600.1 to 809.8], *P<0.0001*) and to MDS and AML patient samples, respectively (700.2 [600.1 to 809.8] versus (vs.) (0.73 [0.04–15.5]; 1.33 [0.03–8.65], *P<0.0001*). There was no significant difference in *CXCR7* expression among patients with MDS, AML, and normal hematopoietic cells (Figure 2A). Among ALL-diagnosed patients, *CXCR7* expression was more pronounced in the T-ALL subtype (T-ALL; 742.2 [629.9 to 793.3] versus (vs.) B-ALL; 637.9 [600.1 to 809.8]; median [minimum – maximum]) (Figure 2B). Noteworthy, we found *CXCR7* expression levels to be positively correlated with bone marrow blast counts (*P = 0.004*) (Figure 3).

### CXCR7 Protein and Gene are More Expressed in Acute T-lymphoid Leukemia Cell Lines

**Figure 4:**
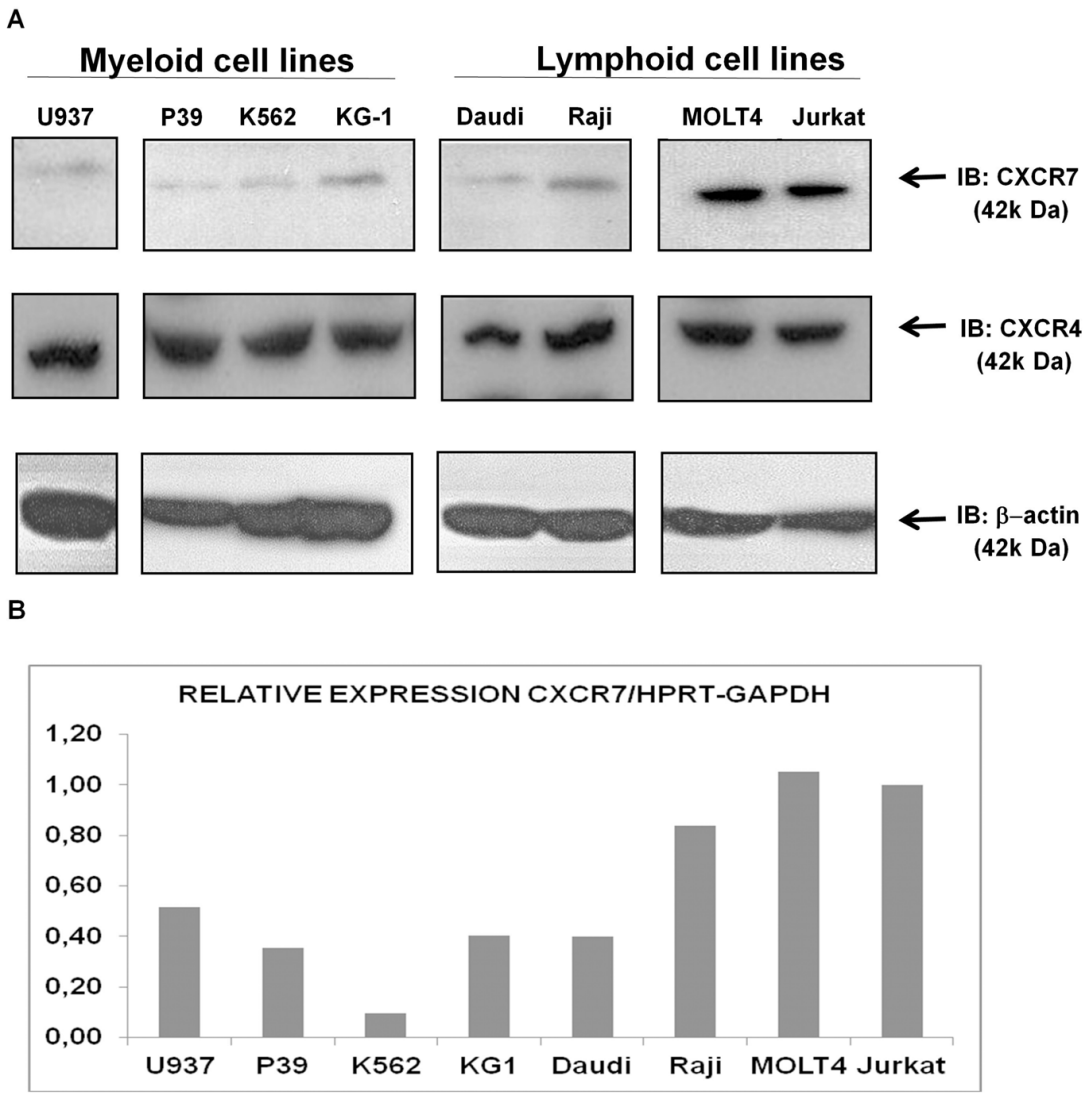
Higher expression of CXCR7 in T-acute lymphoid leukemia lines MOLT4 and Jurkat. A) Western blot analysis of CXCR7 protein levels in myeloid (U937, P39, K562 and KG-1), B-lymphoid (Daudi, Raji) and T-lymphoid (MOLT4 and Jurkat) cell lines. Total cell extracts were blotted with antibodies against CXCR7 (42 kDa), CXCR4 (42 kDa) or β-actin (42 kDa), as a control for equal sample loading, and developed with the ECL Western Blot Analysis System. CXCR7 protein was detectable in all acute leukemia cell lines; however CXCR7 was more expressed in the T-acute lymphoid cell lines MOLT4 and Jurkat when compared to other cell lines. CXCR4 proteins levels were homogeneous in all cell lines analyzed. B) Quantitative expression of *CXCR7* mRNA in leukemic cells lines. mRNA expression levels of *CXCR7* were normalized by *HPRT* and *GAPDH* endogenous control. *CXCR7* mRNA was more expressed in T-acute lymphoid cell lines MOLT4 and Jurkat when compared to other cell lines.

We verified the CXCR7 protein expression levels in myeloid cell lines (U937, P39, K562 and KG-1), B-lymphoid (Daudi, Raji) and T-lymphoid (MOLT4 and Jurkat) cell lines using two methods (Western Blot and Flow Cytometry) and we observed that CXCR7 protein was detectable in all acute leukemia cell lines, CXCR7 however, was more expressed in the T-acute lymphoid cell lines MOLT4 and Jurkat (Figure 4A and Table 2) when compared to other cell lines. This result corroborated the higher gene expression levels found in ALL patients, mainly in the T-ALL subtype. On the other hand, CXCR4 proteins levels were homogeneous in all cell lines analyzed. Furthermore, we observed the CXCR7 gene expression levels by Real-Time RT-PCR and these were similar to the CXCR7 protein expression levels which were more expressed in T-acute lymphoid cell lines MOLT4 and Jurkat when compared to other cell lines, however we noticed a minor difference in the expression of CXCR7 between the B-ALL and T-ALL lines. (Figure 4B).

**Table 2:** Flow cytometry analysis of CXCR7 expression in acute leukemia cell lines.

A. Myeloid	
U937	5%
P39	12%
K562	<3%
KG1	<10%
B. Lymphoid	
Daudi	10%
Raji	54%
MOLT4	98%
Jurkat	95%

Panel A. Myeloid cell lines. Panel B. Lymphoid cell lines. Results are presented as percent of positive cells.

### CXCR7 has Different Cellular Distribution in T-acute Lymphoid Leukemia Cell Lines

**Figure 5:**
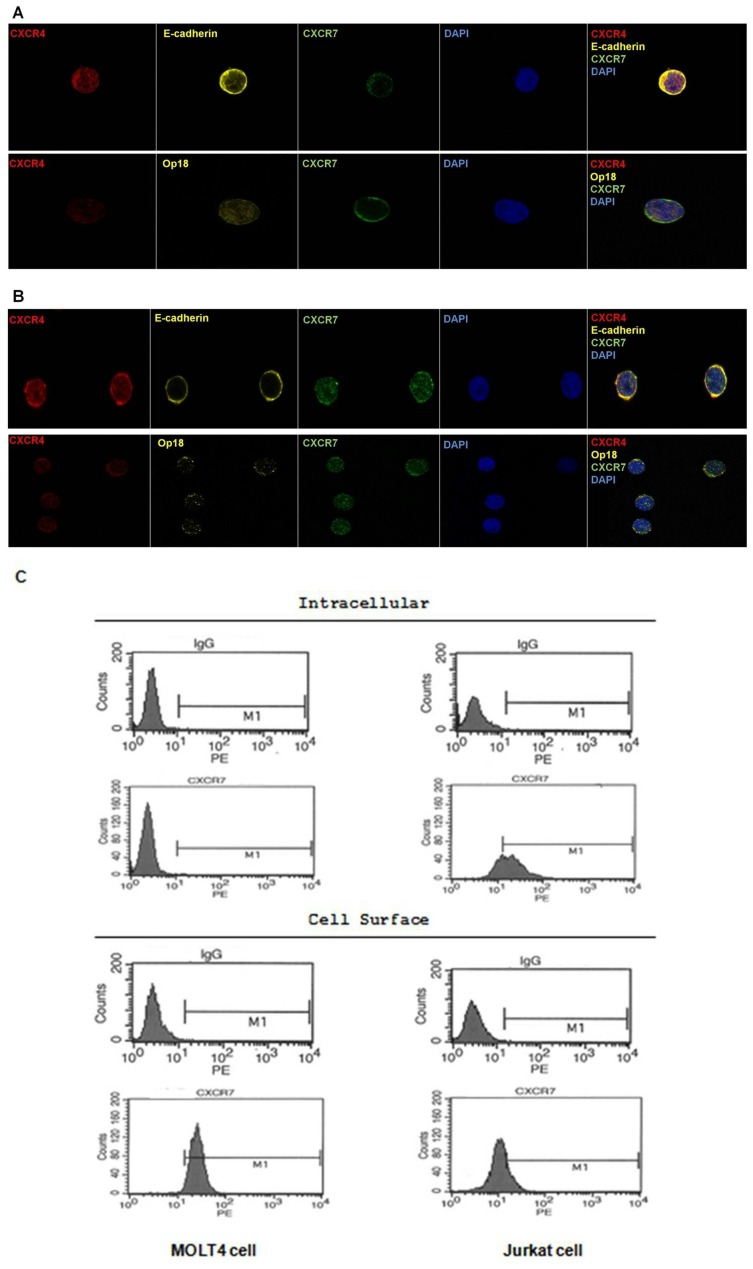
Different localizations of CXCR7 in MOLT4 cells and in Jurkat cells. CXCR4 has the same cellular localization (cell surface and intracellular) in both cell lines. (A–B) Confocal micrographs of MOLT4 and Jurkat cell lines displaying CXCR7 (green) and CXCR4 (red) staining using 63× oil immersion objectives. Appropriated markers for membrane and cytoplasm were used to confirm the localization of these receptors: E-cadherin and Op18 present (yellow), respectively, in the membrane and in the cytoplasm. CXCR7 showed colocalization with these proteins in both cell lines; however CXCR7 was located mainly on the cell surface of MOLT4 cells; unlike, in Jurkat cells, where CXCR7 presented an intracellular and cell surface localization. CXCR4 had same cellular distribution (cell surface and intracellular) in both cell lines. (B) Flow Cytometry, a more quantitative method, confirmed the results observed in confocal microscopy because showed that less than 2% of MOLT4 cells versus 67% of Jurkat cells displayed intracellular CXCR7.

As T-acute lymphoid leukemia cells showed higher CXCR7 expression (gene and protein levels), MOLT4 and Jurkat cell lines were chosen to continue the study. Cellular localizations of both CXCL12 receptors, CXCR4 and CXCR7, were investigated in MOLT4 and Jurkat cell lines by fluorescence and confocal microscopy analysis and also by flow cytometry. Cells were permeabilized in order to enable the detection of internalized receptors. Confocal microscopy analysis evidenced altered cellular distribution of CXCR7 in MOLT4 and Jurkat cell lines: using appropriate markers, E-cadherin and Op18, as membrane and cytoplasm markers, respectively, CXCR7 was observed to be colocalized with both markers in both cell lines, however CXCR7 was located mainly on the cell surface of MOLT4 cells (5A); unlike Jurkat cells, where CXCR7 presented an intracellular and cell surface localization (Figure 5B). CXCR4 had same cellular distribution (cell surface and intracellular) in both cell lines. These results were confirmed by flow cytometry, a more quantitative method, which showed that less than 2% of MOLT4 cells versus 67% of Jurkat cells displayed intracellular CXCR7 (Figure 5C).

### CXCR7 Silencing Decreases T-acute Lymphoid Cell Migration, but does not Modify Proliferation and Apoptosis

**Figure 6:**
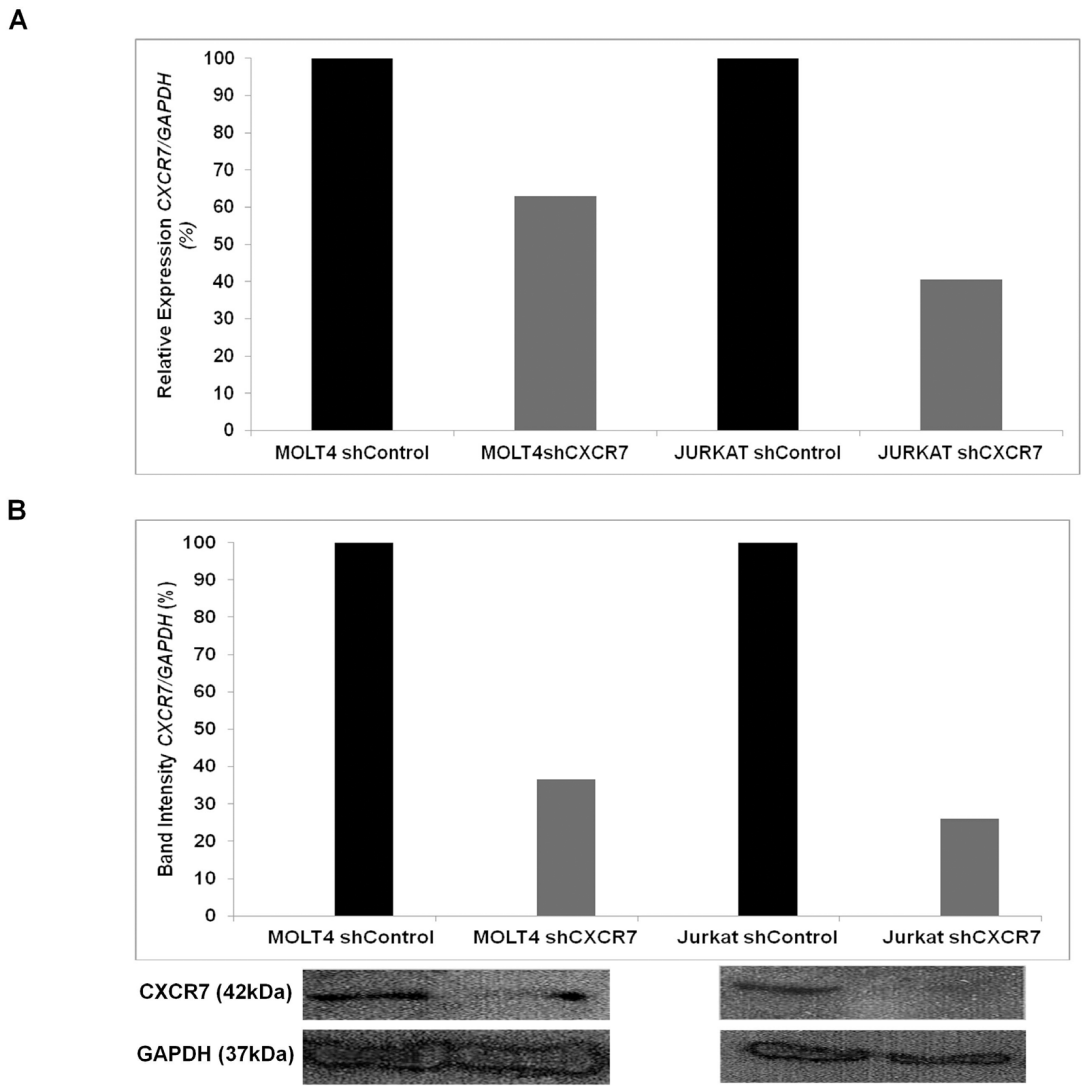
Lentivirus-mediated shRNA targeting *CXCR7* effectively silenced *CXCR7* in MOLT4 and Jurkat cells. A) Quantitative expression of *CXCR7* mRNA in cells relative to the shControl cells. mRNA expression levels of *CXCR7* were normalized by *HPRT* and *GAPDH* endogenous control. Results were analyzed using 2^−ΔΔCT^. *CXCR7* mRNA expression was reduced in MOLT4 cells (41%) and Jurkat cells (63%) when compared with shControl cells. (B) Western blotting analysis of shControl and shCXCR7 cell extracts. The membrane was blotted with antibodies against CXCR7 (42 kDa) or GAPDH (37 kDa), as a control for equal sample loading, and developed with the ECL Western Blot Analysis System. The bar graphs represent the band intensity of CXCR7 protein expression corrected for loading differences based on the corresponding GAPDH bands (UN-SCAN-IT software). Protein levels of CXCR7 were also reduced in MOLT4 cells (63%) and Jurkat cells (74%) when compared with shControl cells.

**Figure 7:**
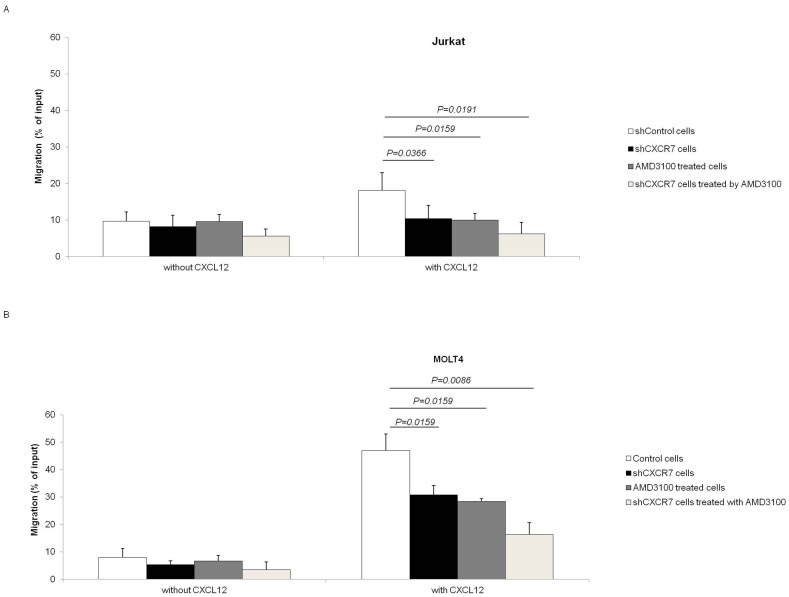
CXCR7 silencing decreases MOLT4 and Jurkat cell migration. Cell migration toward either RPMI with 0.1% BSA and RPMI or 0.1% BSA containing CXCL12 (200 ng/mL) used as negative control and chemoattractant, respectively. After 4 h, the number of migrated cells was counted and was expressed as a percentage of the input, i.e., the number of cells applied directly to the lower compartment in parallel wells. The migration of cells was normalized to 100% +/− sd of triplicates. (A) The CXCR7 silencing resulted in significant changes in MOLT4 chemotactic response (*P = 0.0159*). The inhibition of CXCR4-dependent chemotaxis by its antagonist AMD3100 (1.25 µg/mL) promoted a similar effect (*P = 0.0159*). Moreover, the silencing of CXCR7 plus the treatment with AMD3100 exhibited a synergistic effect in cell chemotactic capacity (*P = 0.0086*). (B) The same effect was observed with Jurkat cells. The CXCR7 silencing (*P = 0.0366*) or the inhibition of CXCR4-dependent chemotaxis by its antagonist AMD3100 (*P = 0.019*) reduced Jurkat chemotactic response. The simultaneous silencing of CXCR7 and treatment with AMD3100 also exhibited a synergistic effect upon cell chemotactic capacity (*P = 0.0191*); *Mann-Whitney* test.

**Figure 8:**
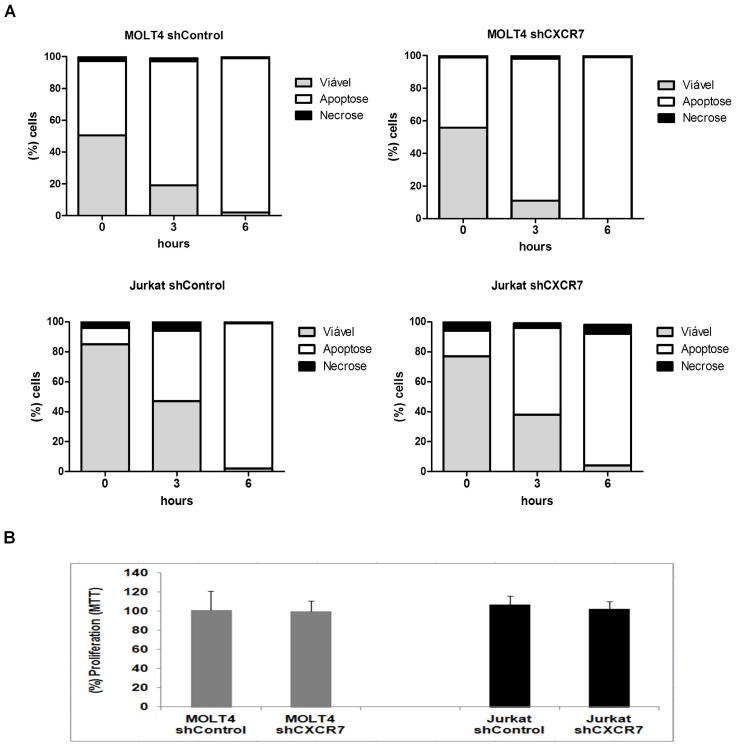
CXCR7 silencing did not modify apoptosis and proliferation of MOLT4 and Jurkat cells. To evaluate whether CXCR7 is important in the process of cell death, control and inhibited CXCR7 cells were exposed to 10 J/m^2^ UV for different periods of time (0, 3, and 6 hours) and apoptosis was detected by flow cytometry using Annexin V/PI staining method. Cell proliferation was determined by MTT assay. Results are shown as mean ±SD of six replicates. No differences in apoptosis rate (A) or proliferation (B) were observed in MOLT4 and Jurkat cell lines.

To observe the role of both receptors in the chemotaxis of and MOLT4 and Jurkat cells, we performed CXCR7 silencing in these cells. Cells were stably transduced with lentivirus-mediated shRNA targeting *CXCR7* (shCXCR7) or lentivirus-mediated shRNA cop *GFP* control (shControl). After selection using puromycin, *CXCR7* mRNA and CXCR7 protein levels were determined by real-time RT-PCR and Western Blot, respectively. The reduction in *CXCR7* mRNA levels and protein levels were normalized to shControl cells. CXCR7 mRNA demonstrated a reduction of 41% and 63%, respectively, in MOLT4 and Jurkat cell lines (Figure 6A). Densitometry analysis of the Western Blot assay, by gel analysis software (UN-SCAN-IT), showed a 63% and 74% reduction of protein levels in MOLT4 and Jurkat cells, respectively (Figure 6B), after silencing of CXCR7. In addition, we performed the inhibition of CXCR4-dependent chemotaxis with the antagonist AMD3100 (1.25 µg/mL). Transwell-chemotactic assay revealed that in both MOLT4 and Jurkat cell lines, there was a significant reduction in shCXCR7 cell migration compared to shControl cells (*P = 0.0159 and P = 0.0366,* respectively). The inhibition of CXCR4-dependent chemotaxis by its antagonist AMD3100 promoted a similar effect when compared to shControl cells (MOLT4, *P = 0.0159* and Jurkat, *P = 0.0119*). Moreover, the silencing of CXCR7 plus the treatment with AMD3100 exhibited a synergistic effect in cell chemotactic capacity (*P = 0.0086; P = 0.0191* respectively; Figure 7). Moreover, shCXCR7 cells did not display significant differences in proliferation rates, as demonstrated by MTT assay, or in apoptosis induction, as demonstrated by Annexin V positivity, suggesting that, in these cells, CXCR7 has a mainly chemotactic-controlling role (Figure 8A–B), and potentiates CXCR4 response.

### Expression of CXCR7 in Peripheral Blood Leukocytes

**Figure 9:**
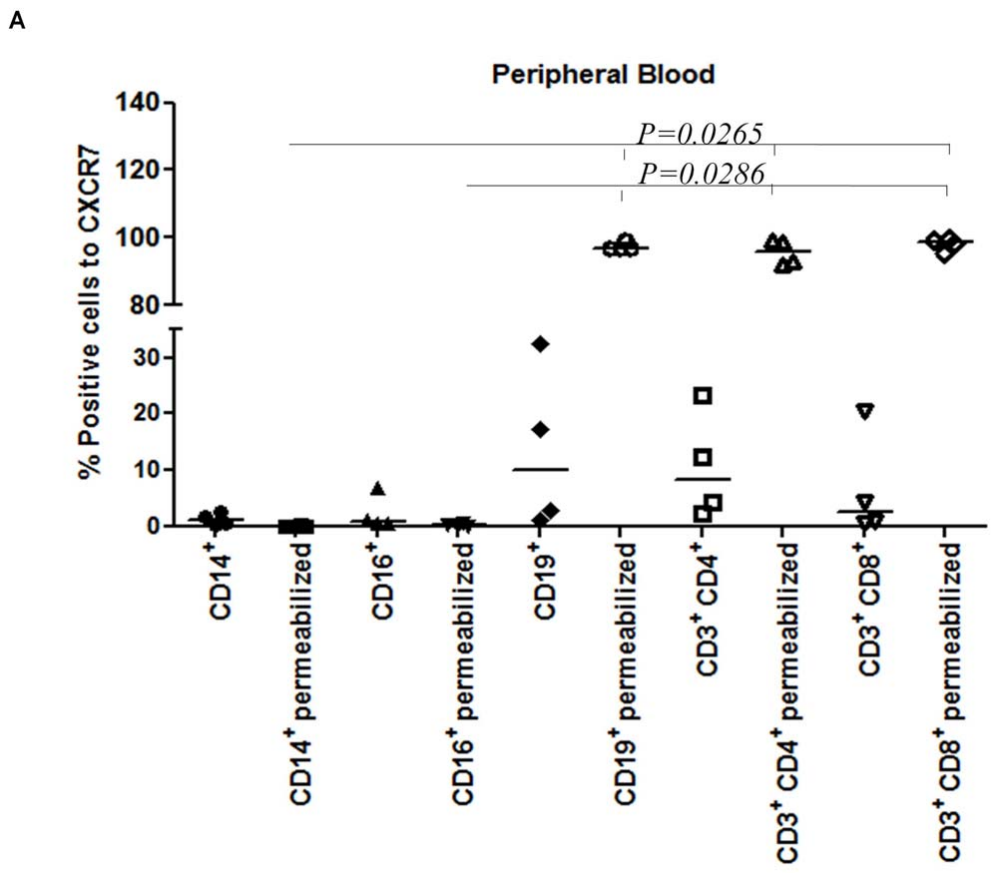
High CXCR7 expression of peripheral blood and bone marrow lymphocytes. (A) CXCR7 is expressed in peripheral blood leukocytes, however an increase in CXCR7 cell surface expression was observed in lymphocytes compared to monocytes and neutrophils. This difference was more apparent and significant when the cells were permeabilized (lymphocytes vs. monocytes, *P = 0.0265* and lymphocytes vs. neutrophils, *P = 0.0286*) showing that the localization of this receptor is mainly intracellular in B-lymphocytes, CD4^+^ T-lymphocytes and CD8^+^ T-lymphocytes; *Mann-Whitney* test.

We next evaluated CXCR7 protein expression in definitive (adult) human leukocyte subsets by flow cytometry. CXCR7 was expressed in peripheral blood leukocytes, however, an increase in CXCR7 cell surface expression was observed in lymphocytes compared to monocytes and neutrophils. This difference was more significant when the cells were permeabilized (lymphocytes vs. monocytes, *P = 0.0265* and lymphocytes vs. neutrophils, *P = 0.0286*) showing that the localization of this receptor is mainly intracellular in B-lymphocytes, CD4^+^ T-lymphocytes and CD8^+^ T-lymphocytes (Figure 9).

## Discussion

Chemokines are considered to play a role in cancer migration and growth. Previous studies have shown a significant correlation between chemokine receptor status in human cancers and prognosis and/or metastases in a variety of malignant tumors such as T-cell leukemia (CCR4), hepatocellular carcinoma (CCR6), gastric carcinoma (CCR7), renal cell carcinoma (CXCR3), ovarian cancer (CXCR4), osteosarcoma (CXCR4), colorectal cancer (CCR7 and CXCR4), and malignant melanoma (CXCR3 and CXCR4) [22]. In this report we investigated the expression of the novel chemokine receptor CXCR7 in myelodysplastic syndromes and acute leukemias. We found that CXCR7 was expressed at higher levels in bone marrow cells from acute leukemia patients and the highest expression observed in ALL patients was confirmed at the protein level. The increased expression of CXCR7 in lymphoid leukemia cells detected in this study is a phenomenon observed in a variety of solid tumors such as brain, prostate, lung, breast, prostatic, renal and glioma cells in which the increased expression of CXCR7 has been positively correlated with aggressive tumor behavior [15], [33]. In agreement with our findings of a lower *CXCR7* expression in normal BM cells, Berahovich and co-workers detected neither CXCR7 gene or protein expression in human and mouse leukocytes [44], hence, the biological function that CXCR7 plays could be cell type specific [38]. Despite CXCR7 only being involved in the cell proliferation of solid tumors [34], [45], a contribution of this receptor in migration of lymphoid leukemia cell lines was indeed herein, unveiled. In accordance to our data, Tarnowski et al. [46] demonstrated the participation of CXCR7 in migration/adhesion of malignant hematopoietic cells but not in the proliferation status of these cells. A crucial function of CXCR7 in controlling the migration of CXCR4^+^ cells towards CXCL12 gradients by sequestration of excess CXCL12, has been reported. Excess of chemokine may result in desensitization of the chemokine receptor and cell migration blockage. Therefore, CXCR7 sequestration of CXCL12 could be of vital importance for the migration of CXCR4^+^ cells [16], [31], [47], [48]. Similarly, a recent paper by Cruz-Orengo and colleagues showed that CXCR7 expression in endothelial cells from inflamed central nervous system tissue was essential in controlling CXCR4^+^ leukocyte entry into the tissue by scavenging and controlling the CXCL12 gradient [16], [49].

Our data further suggests that the presence of CXCR7 may also be important in potentiating the migration induced by CXCL12/CXCR4 binding in T-acute lymphoid leukemic cells. CXCR7 potentiation of CXCL12/CXCR4 signaling has been suggested due to the heterodimerization of both receptors [6], [14], [31], [50]. Heterodimerization of CXCR4 and CXCR7 resulted in the attenuation of classical CXCL12-mediated Gi-activated signaling as measured by classical Gi activation assays that monitor adenylyl cyclase inhibition. Receptor dimerization/oligomerization has emerged as a key paradigm in G protein-coupled receptor (GPCRs) biology and has been implicated in almost all aspects of GPCR function, including intracellular trafficking receptor internalization, pharmacological inhibition, and signal transduction. Despite the challenge of ascertaining functional consequences of receptor heterodimerization, heterodimerization has been demonstrated to be capable of completely changing the activated signaling pathways as well as the trafficking of the receptors [3]. In the case of the CXCR4-CXCR7 heterodimer, Décaillot et al. [3] observed a switch in signaling pathways that were induced downstream to CXCL12 stimulation, a decrease in G protein-dependent signaling, and an increase in arrestin recruitment and signaling. Lymphocytes have been used as cell models for CXCR7 studies, the results however are contradictory [38]. An initial study showed a high expression of CXCR7 in human T-lymphocytes [21], supported by our data of high *CXCR7* gene expression in bone marrow and lymphoblasts of T-ALL patients, T-lymphocytes in peripheral blood of healthy donors and CXCR7 protein in T-acute lymphoid leukemia cell lines. However, Hartmann et al. reported very low levels of CXCR7 in normal T-cells by flow cytometry [11]. This difference could be related to the different protocols used, as in Hartmann’s study the cells were not permeabilized [11] and CXCR7 was only detected on the cell surface. Despite its high affinity to CXCL12, the role of CXCR7 in CXCL12-dependent cell motility and chemotaxis is currently under controversial debate. In lymphocytes, one study has suggested that CXCL12 signals through CXCR7 on primary T-cells and that CXCR7 co-participates with CXCR4 in lymphocyte motility [21], whereas a second group observed no contribution of CXCR7 in T-cell migration [21]. In agreement with this second group, Hartmann et al. detected no effect of CXCR7-blocking mAb or the CXCR7 antagonist CCX733 on this CXCL12-triggered motility [11]. However, our study shows that CXCR7 contributes for T-ALL cells migration induced by CXCL12. In addition, our results suggest that CXCR7 expression may be important in leukemogenesis as CXCR7 expression correlated positively with the percentage of bone marrow blasts. Herein, a different CXCR7 cellular localization in MOLT4 and Jurkat cell lines was observed and this localization could be related to cell chemotactic capacity, since MOLT4 cells demonstrated a higher migration compared to Jurkat cells. However, CXCR4 and CXCR7 expression has been reported to have an intracellular location in other cancers such as gallbladder and pancreas without CXCL12 stimulation [15], [23]. The low percentage of Jurkat cell migration to CXCL12 here observed (∼20%) has also been described by Ottoson et al. [51], however, Butler et al. observed that a higher number of cells were capable of migrating toward the chemoattractant CCL12 [52]. Despite of a biological conclusion, based on the comparison of different cell lines, being difficult to assert, we found MOLT4 and Jurkat cells to be interesting models, since both cell lines were obtained from patients with T-acute lymphoid leukemia and were submitted to the same experimental conditions.

Most studies addressing the involvement of chemokines and their receptors in the tropism of leukemic cells have focused on the interaction of CXCL12 and its receptor CXCR4. Given that BM stromal cells are major producers of CXCL12, and CXCR4 expression is thought to be higher on BM-residing blasts than on circulating blasts, CXCR4/CXCL12 interactions are likely to facilitate the retention of blasts in the BM [18]. As ALL blasts are known to have high levels of CXCR4 and we also detected high levels of CXCR7, this receptor could possibly potentiate the homing and retention of these blasts in BM. Thus, an inhibition of both receptors could decrease the homing of leukemic blasts in the BM microenvironment and be associated with a better response than the blocking of a single receptor [15]. Therefore, future studies will be necessary to reach consistent conclusions regarding the role of CXCR7 in acute lymphoid leukemia pathogenesis and prognosis.
