# Correction: CXCR7 Is Highly Expressed in Acute Lymphoblastic Leukemia and Potentiates CXCR4 Response to CXCL12

**DOI:** 10.1371/journal.pone.0091365

**Published:** 2014-03-14

**Authors:** The PLOS ONE Staff

The name of the first author is incorrectly represented in the citation. The correct citation is: Melo RCC, Longhini AL, Bigarella CL, Baratti MO, Traina F, et al. (2014) CXCR7 Is Highly Expressed in Acute Lymphoblastic Leukemia and Potentiates CXCR4 Response to CXCL12. PLoS ONE 9(1): e85926.doi:10.1371/journal.pone.0085926.
